# Social Determinants Associated with Health Care Empowerment Among University Students: A Cross-Sectional Pilot Study

**DOI:** 10.3390/ijerph23081022

**Published:** 2026-08-04

**Authors:** Miluska Vega-Guevara, Felipe Aguirre-Chávez, Rosa Millones Rivalles, Vicenta Irene Tafur Anzualdo

**Affiliations:** Institute of Research (ININ), Marcelino Champagnat University, Lima 15039, Peru; miluska.vega@umch.edu.pe (M.V.-G.); faguirre@umch.edu.pe (F.A.-C.); rosa.millones@umch.edu.pe (R.M.R.)

**Keywords:** social determinants, empowerment, health care, university students, ECSU

## Abstract

**Highlights:**

**Public health relevance—How does this work relate to a public health issue?**

- Social determinants such as income level, housing conditions, and family structure were associated with variations in health care empowerment among university students.
- Understanding these associations is particularly relevant in Latin American higher education settings, where social and economic inequalities may influence health-related behaviors and access to care.

**Public health significance—Why is this work of significance to public health?**

- The study provides evidence from Peru on the relationship between social determinants and health care empowerment, a topic that remains underexplored in university populations.
- The adapted ECSU instrument demonstrated adequate psychometric properties, supporting its use in future research on empowerment and self-care among young adults.

**Public health implications—What are the key implications or messages for practitioners, policy makers and/or researchers in public health?**

- Universities may strengthen student well-being by implementing health promotion strategies that consider socioeconomic and family-related vulnerabilities.
- Future studies using broader and probabilistic samples are needed to confirm these findings and support evidence-based public health interventions.

**Abstract:**

Health care empowerment is essential for self-care, informed decision-making, and appropriate use of health services among university students. This cross-sectional pilot study examined the association between selected social determinants of health and health care empowerment among university students in Peru. A sample of 336 undergraduate and graduate students from six universities completed an online survey that included the Empowerment Scale for Health Care Empowerment in University Students (ECSU). The instrument demonstrated adequate psychometric properties (Cronbach’s α = 0.859; McDonald’s ω = 0.835). Descriptive analyses and ordinal logistic regression were conducted to identify factors associated with health care empowerment and its dimensions. Income level, family type, and housing situation were associated with overall empowerment. Lower income levels and extended family structures were associated with lower empowerment levels, while university type and educational level were associated with specific dimensions related to decision-making and the ability to empower others. The explanatory capacity of the models was modest (pseudo-R^2^ = 5.0–12.8%). These findings provide preliminary evidence on the relationship between social determinants and health care empowerment in university students. However, results should be interpreted with caution due to the cross-sectional design and convenience sampling. Future studies using probabilistic sampling and longitudinal designs are needed to confirm these associations.

## 1. Introduction

Income level, housing situation, and family type are significant social determinants that may be associated with variations in health care empowerment, especially in the young population. According to the World Health Organization (WHO), these determinants are part of the “conditions in which people are born, grow, live, work, and age,” which are shaped by the distribution of power and resources at global, national, and local levels [1]. Identifying these social determinants, their relationships, and the major risk factors requires first defining what is meant by social determinants. Social determinants include aspects such as age, sex, place of origin, family type, housing situation, educational level, occupation and income level [2]. These factors make up the social profile that influences the health status of individuals.

This set of concrete and specific conditions and circumstances in which people are born and develop are known as social determinants. Other frameworks, such as those from the CDC, classify them into domains including economic stability, education, health care access, social context, and neighborhood environment [3]. All aspects of human and social life are influenced by social determinants. However, it is important to recognize that not all are of equal importance, as some may represent greater risks due to their uniqueness and unrepeatability.

Although a substantial body of international research has examined the influence of social determinants on health outcomes, the specific relationship between these determinants and health care empowerment remains insufficiently explored, particularly within university populations. Existing studies have predominantly focused on isolated factors—such as socioeconomic status or access to health services—without adopting an integrated analytical approach to empowerment as a multidimensional construct.

This limitation is more pronounced in Latin American contexts, including Peru, where empirical evidence addressing the interaction between structural inequalities and empowerment processes in young populations is still limited. Consequently, the lack of context-sensitive research constrains the development of evidence-based interventions and institutional policies aimed at strengthening self-care, health literacy, and autonomous decision-making among university students, a population facing complex and intersecting social, economic, and educational challenges.

**Figure 1:**
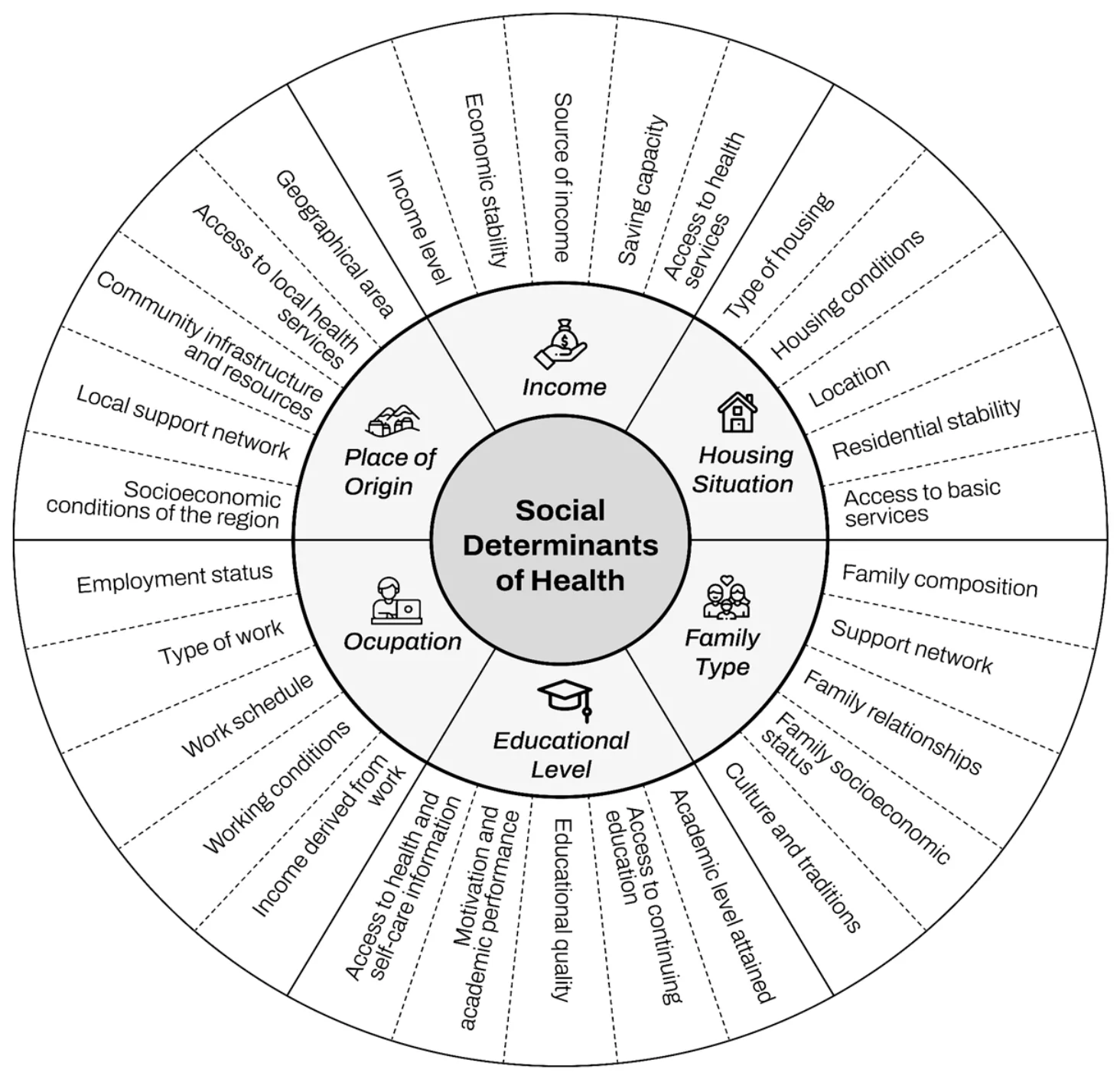
Classification of Social Determinants of Health.

In Figure 1 (developed by the authors), a comprehensive framework is presented to understand the social determinants of health affecting university students, categorized into six key domains: Income, Housing Situation, Family Type, Educational Level, Occupation, and Place of Origin. Each domain includes specific factors such as economic stability, housing conditions, family support networks, access to education, employment status, and regional resources, highlighting how these interconnected elements shape students’ health care empowerment. By identifying areas of vulnerability, such as limited income, unstable housing, or lack of educational resources, the diagram emphasizes the need for targeted interventions to promote self-care and improve health outcomes in young populations. This holistic perspective aligns with contemporary approaches that underscore the intersectionality of social, economic, and environmental factors in health care empowerment [4].

The empirical findings of this study support this framework, particularly highlighting income level, housing situation, and family type as the most significant determinants associated with health care empowerment among university students. Educational contexts involve multiple interacting physical, psychological, and pedagogical factors that influence student development and well-being [5,6]. However, in the present study, the analysis focuses specifically on measurable social determinants such as income level, housing situation, and family type, which showed significant associations with health care empowerment. 

**Figure 2:**
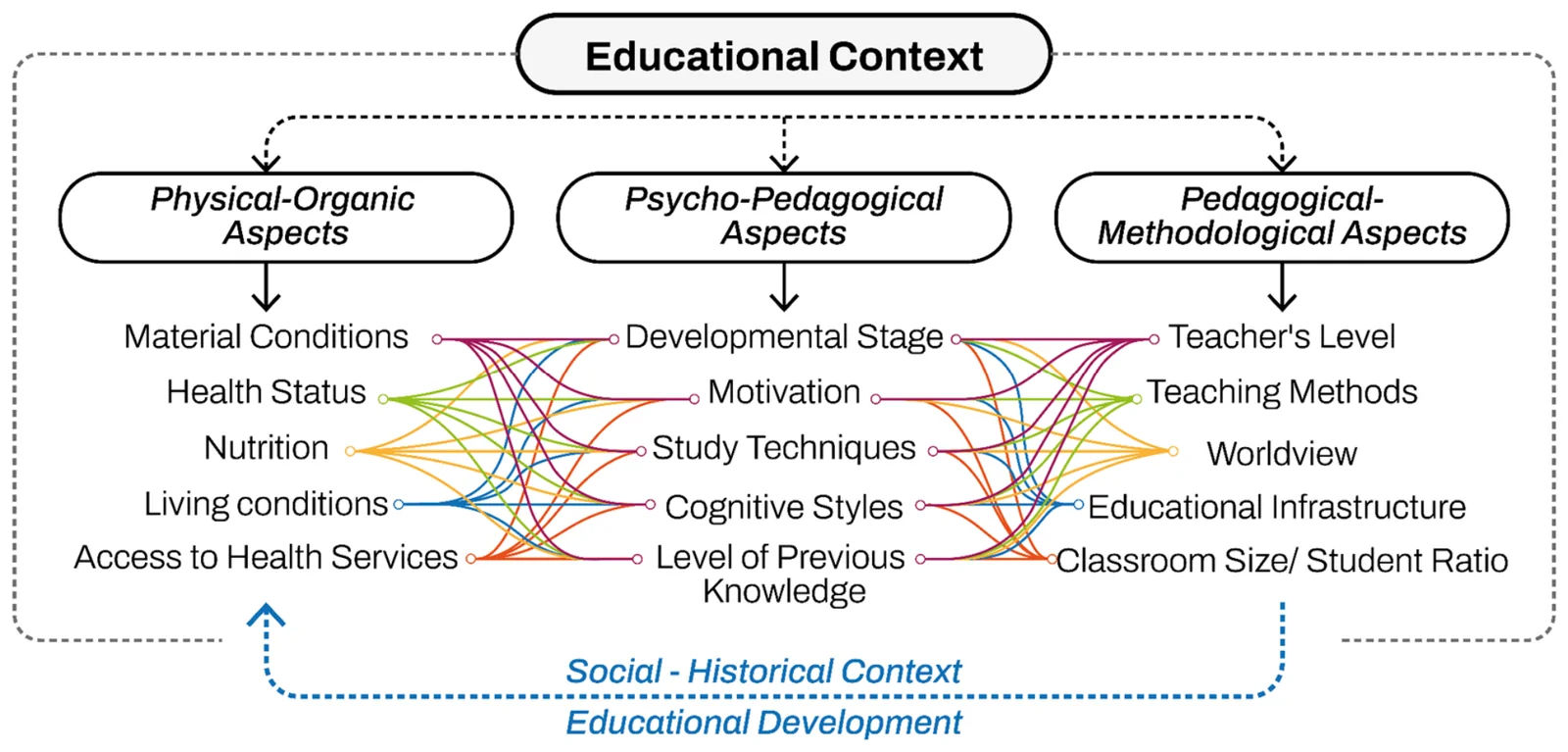
Social Determinants of Health in Educational Settings: A Comprehensive Framework. Different colored lines are used solely to improve visual clarity and distinguish the connections among the elements; they do not represent different types or strengths of relationships.

In Figure 2 (developed by the authors), an integrated model is presented that organizes the social determinants of health in the educational context, emphasizing the interrelation between physical-organic, psycho-pedagogical, and pedagogical–methodological aspects. This structure highlights how material conditions, psychological development, and educational resources do not operate in isolation but are dynamically interconnected, creating a comprehensive environment that can positively or negatively impact students’ health and well-being. Thus, the figure underscores the importance of understanding these areas as interdependent elements whose combined impact can either enhance or limit health care empowerment within educational settings.

The results obtained in this study support this integrated perspective, showing that social and educational factors, particularly income level and type of university, were significantly associated with dimensions such as positive attitude and decision-making capacity.

First-generation university students are defined as those whose parents did not complete higher education, and they represent a vulnerable subgroup within the academic community. Research shows that they are more likely to experience financial strain, limited access to social and academic support networks, and lower levels of self-efficacy compared to continuing-generation peers [5,6,7]. Parental educational attainment functions as a clear social barrier that constrains access to key resources, thereby limiting the development of self-efficacy and health-related agency. These disadvantages may hinder their ability to engage in self-care and to develop health care empowerment, making them a population of particular concern in studies addressing social determinants of health. These findings are consistent with the results of the present study, where social determinants such as income level and type of university were identified as significant factors associated with variations in health care empowerment.

Health care empowerment is defined as the set of feelings of control, self-efficacy, coping and capacity for change that people develop in relation to their health. This implies a posture that determines the participation and self-management of the person in relation to his/her health care. Empowerment can have various facets, but in this research, psychological empowerment and social empowerment were the main ones considered [7]. 

Psychological empowerment has been described as the perception of control, competence, and the capacity to influence one’s environment [4]. On the other hand, social empowerment implies an increase in the capacity of individuals and collectives to define, analyze and act on their own problems [8].

**Figure 3:**
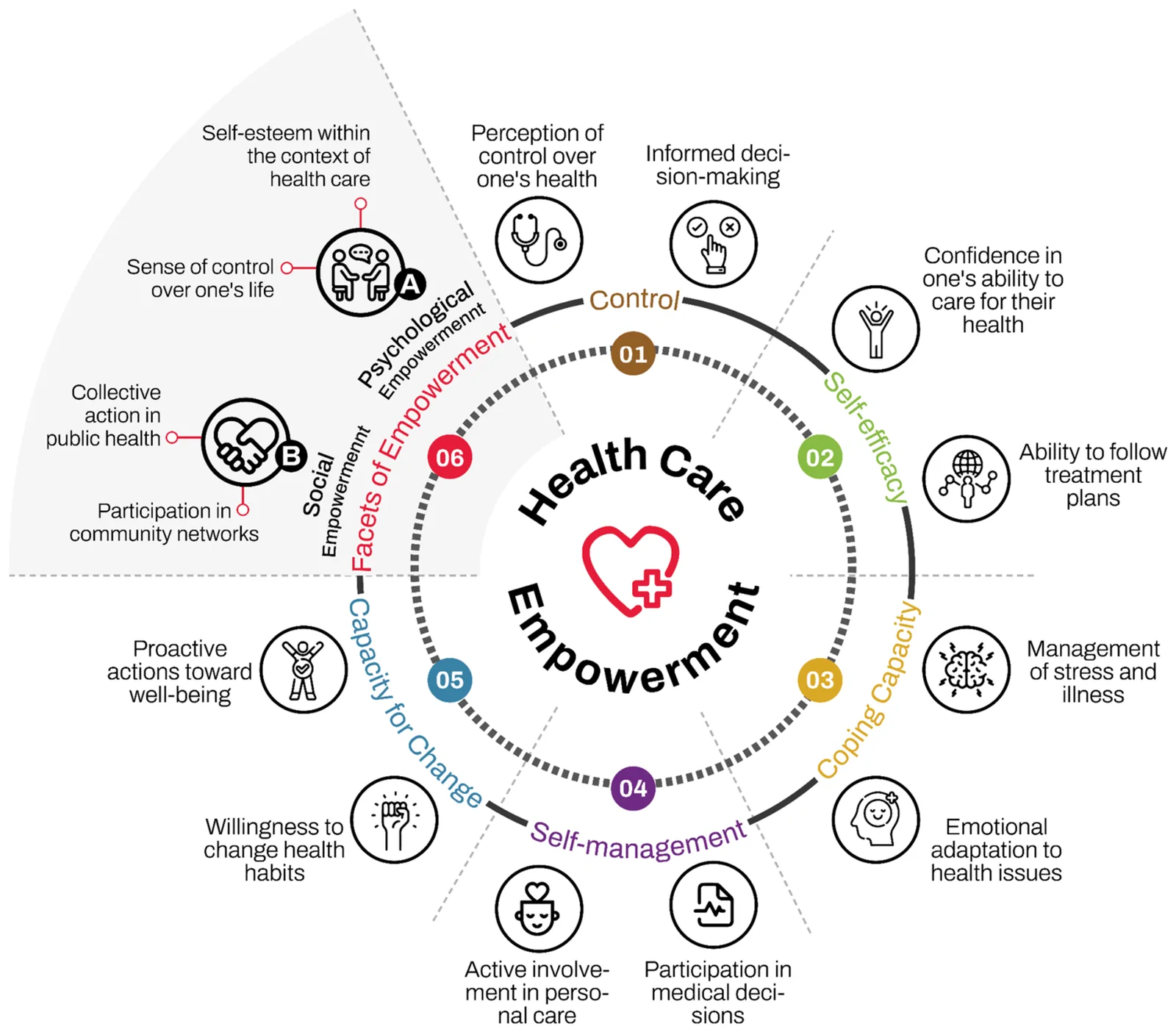
Dimensions and Facets of Health Care Empowerment.

In Figure 3 (developed by the authors), a schematic representation is presented showing the key dimensions of health care empowerment, structured into six interrelated categories: control, self-efficacy, coping capacity, self-management, capacity for change, and the facets of empowerment (psychological and social). The figure illustrates how personal resources, such as perceived control, informed decision-making, confidence in one’s abilities, and emotional adaptation, interact dynamically with contextual factors such as community participation and collective action. This conceptualization emphasizes that health empowerment is an evolving and multifactorial process, rather than a static condition. Moreover, it helps identify points of intervention at both individual and collective levels, which is essential for designing comprehensive strategies aimed at strengthening autonomy, shared responsibility, and active participation in the promotion of health and well-being.

These dimensions directly informed the dependent variables examined in the regression models (Section 3.2), where income level and university type were found to be differentially associated with self-efficacy and decision-making confidence. Income level, housing situation, and family type are significant social determinants that may be associated with variations in health care empowerment, particularly among young populations. Identifying these determinants, their interrelationships, and the groups most at risk requires first establishing what is meant by social determinants and then determining which factors represent the greatest risks.

Social determinants are made up of factors such as age, sex, place of origin, type of family, housing situation, educational level, occupation and income level; all of which form the social profile that influences people’s health status. This set of specific conditions and circumstances into which individuals are born and develop constitutes the social determinants [9]. There is no object, field, or dimension of social human life outside the scope of social determinants, although not all have the same importance; some may represent greater risks and are unique and unrepeatable. 

In the educational process, the social determinants make up the physical-organic aspects (material conditions of life and activity, nutritional and health status of the student), psycho-pedagogical (developmental stage and psychological particularities of the student, state and level of previous knowledge, cognitive styles and rhythms, motivation, self-motivation and study techniques), pedagogical–methodological (conception of the world, personality characteristics, scientific-professional level, teaching styles and methods of the teacher) and, naturally, the concrete conditions in which the educational process takes place (school environment, number of students per classroom, educational infrastructure, didactic means and materials). When these mediational factors are overlooked or are not adequately specified in the concrete particularities of each of these elements and their reciprocal intertwining, they can easily lead to a formalistic and abstracted vision of the educational process. Hence, the social–historical context and human development are dynamic forces of reciprocity: the social–historical context determines, conditions and mobilizes the formative process, and the latter, in turn, contributes to the impulse of this development [10].

Empowerment in health care is the set of feelings of control, self-efficacy, coping capacity and capacity for change that people develop with respect to their health, that is, the position that determines their participation and self-management in health care. Empowerment, then, could have multiple edges, but the research basically considered psychological empowerment and social empowerment. Psychological empowerment contributes to a feeling of greater control over one’s own life that individuals experience through belonging to different groups; and social empowerment increases the capacity of individuals and groups to define, analyze and act on their own problems [11].

Psychological and social aspects constitute interactive and fundamental links in human development. According to Vygotsky, they are objects of education and can be shaped through the educational process. Education, as a process, is the most appropriate means to foster people’s psychological and social development. It contributes to the objective understanding of one’s own reality and, on that basis, promotes not only proactive thinking but also the development of concrete, responsible, and forward-looking actions in health care. Ultimately, this process enables people to increase control over their health and thus improve it [12].

**Figure 4:**
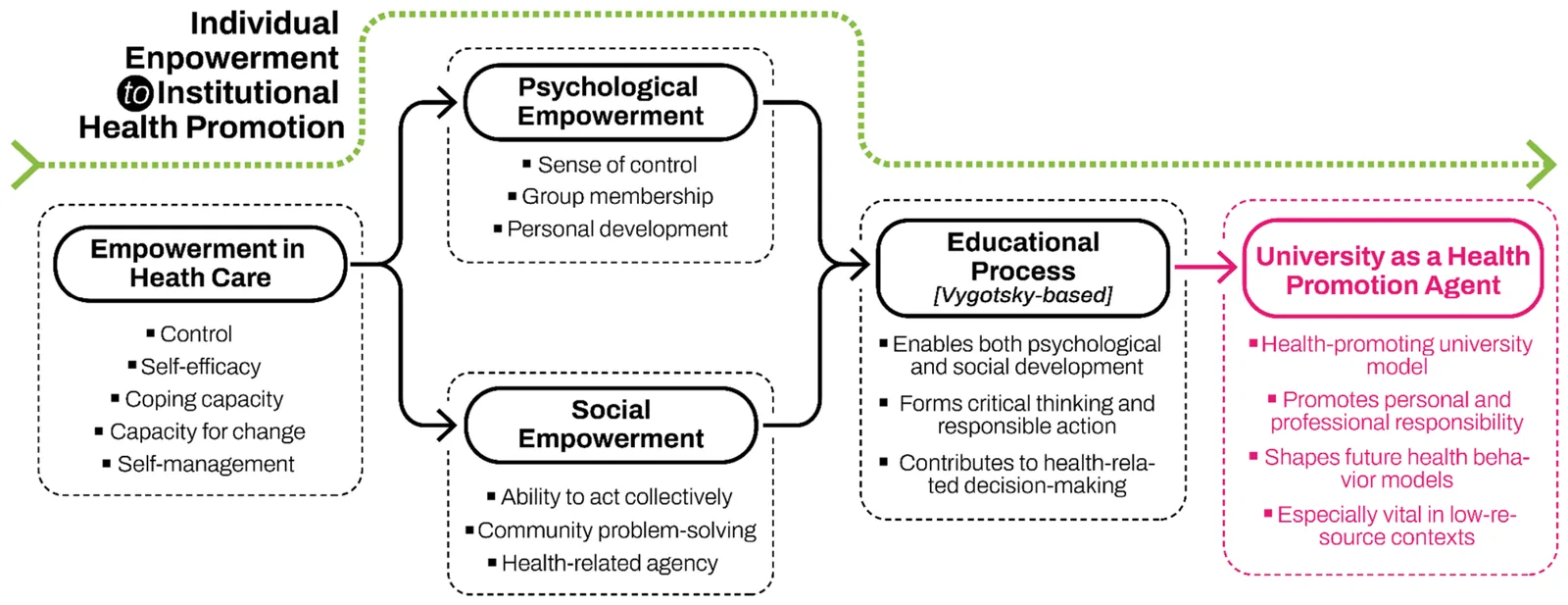
Pathway from Individual Empowerment to Institutional Health Promotion through Higher Education.

Figure 4 (developed by the authors), illustrates the flow from individual empowerment to institutional health promotion, emphasizing the interrelations among various levels of personal, social, and educational development. The process begins with empowerment within the domain of health care, where capacities such as control, self-efficacy, change management, and self-regulation are reinforced. This empowerment extends into psychological and social dimensions, fostering a sense of control, group membership, and personal development, as well as collective action, community problem-solving, and health-related agency. Both dimensions converge within an educational process grounded in Vygotskian theory, which strengthens psychological and social development, promotes critical thinking and responsible action, and supports informed health-related decision-making. This relationship culminates in the institutional relevance of the university as a medium for health promotion, embodied in a model that fosters personal and professional responsibility, shapes future health behaviors, and gains particular importance in resource-limited contexts.

In contexts with limited resources, the role of educational agents, including universities, becomes increasingly important. Universities should function as central agents of health promotion and development. As part of their mission to train competent professionals, they must implement actions that empower students and foster responsibility for their health care. Concretely, this may include providing free or subsidized on-campus health services for low-income students, flexible attendance or online learning options for those with caregiving or work responsibilities, and targeted programs to strengthen health literacy among first-generation students. 

In this sense, universities integrate health promotion into their educational and institutional projects, with the aim of fostering human development and improving the quality of life of students and staff. At the same time, they prepare individuals to act as role models and promoters of healthy behaviors within their families, future workplaces, and society at large [13].

Health promotion, health care, and education as values, processes, and tools have reciprocal relationships. According to the theoretical model of self-care proposed by Orem, every human being can care for and learn to care for themselves. However, achieving this requires relevant knowledge, practical skills for action, and motivation to engage in such care. In any case, education is an effective means of raising awareness of empowerment and making healthcare activities effective. Self-care is reflected not only in people’s behavior in situations that affect their development and health, but also prospectively in health-promoting and sustainable actions [14]. For example, women with higher levels of empowerment tend to have lower barriers to health care, and this increases in rural areas, and cultural diversity, citizen participation, and empowerment are determining factors in the effectiveness of actions aimed at ensuring public health. 

Young university students are a vulnerable population that should be investigated in a particular way, to create and evaluate intervention programs according to their needs. Supporting youth health care through multiple mechanisms is crucial, since addressing social determinants as risk factors in health–disease processes is a complex challenge. These risks may compromise autonomous decision-making in areas such as sexuality, reproduction, access to timely information, and employment opportunities, thereby affecting health care empowerment [15]. 

Another important risk factor, also linked to the above, is nutritional vulnerability. Studies conducted at the Universidad Surcolombiana revealed that most students exhibited risk behaviors associated with poor nutrition and inadequate health care, for example, excessive consumption of sugars and fats or difficulties in stress management.

In Latin American contexts, evidence shows both positive and negative patterns: while some studies report healthy sexual behaviors among medical students, others highlight that health promotion systems remain insufficient [16].

Indeed, globalization and technological development have generated economic growth and cultural changes in people’s daily lives, they have also widened social gaps, increased poverty, exclusion, and inequality, and contributed to the epidemiological transition of diseases. Addressing these challenges requires not only welfare policies aimed at consequences but also strategies grounded in self-efficacy and social empowerment, enabling people to assume responsibility for their health care.

Despite the growing interest in social determinants of health, there is still limited empirical evidence examining the extent to which key structural factors—such as income level, family structure, and type of university—are associated with variations in health care empowerment among university students. This gap is particularly relevant in the Peruvian context, where the interaction between social inequality and empowerment processes remains underexplored within higher education settings.

Addressing this limitation is not only important from an applied perspective but also contributes to advancing theoretical understanding of empowerment as a context-dependent and multidimensional construct. By identifying how these determinants are associated with differential levels of empowerment, the study provides a basis for developing more context-sensitive institutional strategies aimed at reducing inequalities and strengthening student well-being.

Finally, the identification of social determinants associated with health care empowerment in university students constitutes a current issue aligned with the Sustainable Development Goals. It highlights the need to strengthen links between social and educational organization and health care in this vulnerable population segment. The evidence obtained can guide the implementation of actions aimed at reducing risk factors and improving student health care, thereby positioning the university as an educational entity that promotes health within its area of influence [17]. All figures presented in this article are original elaborations by the authors, based on the theoretical frameworks reviewed.

This study aims to examine how social determinants of health are associated with health care empowerment among university students, with particular emphasis on vulnerable groups such as first-generation students, and to propose a conceptual model that links individual empowerment with institutional health promotion. Identifying these determinants can provide universities with practical guidelines to design preventive interventions and policies that strengthen student self-care, access to health services, and overall well-being. Specifically, the objectives are to:

- Identify the main social determinants of health that may represent risk factors for empowerment
- Conceptualize the psychological and social dimensions of health care empowerment in the educational context
- Develop author-based conceptual figures that illustrate the pathways linking social determinants, empowerment, and institutional health promotion, and critically assess the internal validity and consistency of the proposed conceptual frameworks
- Contribute exploratory preliminary findings to inform the design of targeted interventions and institutional policies aimed at strengthening student self-care and health promotion within universities.

This study contributes to the literature by advancing an integrated analytical framework that examines the relationship between social determinants and health care empowerment as a multidimensional construct within university populations. Unlike previous studies that have addressed these variables in isolation, this research adopts a comprehensive approach that allows for the identification of combined and context-specific risk factors.

Furthermore, by providing empirical evidence from a Latin American context, the study extends existing knowledge beyond predominantly high-income settings and offers insights into how structural inequalities are associated with empowerment processes in higher education environments. This contribution is particularly relevant for refining theoretical models of empowerment and informing context-sensitive institutional strategies.

## 2. Materials and Methods

The research corresponds to a quantitative approach with a non-experimental, cross-sectional design, specifically descriptive-correlational in scope. This design was chosen because it allows the identification of associations between social determinants of health (independent variables) and health care empowerment (dependent variable) without manipulating the study conditions. The cross-sectional nature of the study made it possible to collect data at a single point in time from a convenience sample of university students, enabling the exploratory analysis of associations relevant to this population. Given the pilot nature of this study, results should be interpreted with caution and cannot be generalized beyond the sample.

The sample consisted of 336 students from five universities in the city of Lima and one university in the province, who agreed to participate voluntarily in the research. 

Fifty-five percent of the students came from national universities and 45% from private universities. Approximately 75% were female, while 19% were male; in addition, a separate item collected information on gender identity to differentiate it from sex assigned at birth. In the sample there was a slight predominance of students aged 19 to 30 years (44%); 56% of students came from the city of Lima and the remaining 44% from the provinces [18].

As for family type, more than 60% of students belonged to nuclear families, about 19% to extended families, the remaining 13% to single-parent or unipersonal families. The housing situation of 85% of the participants is urban and 15% rural. In total, 63% of the sample only studied and less than 30% had a formal job, with the majority having an income of less than 2500 soles per month: 32% of participants had a monthly income of less than 900 soles, 19% between 900 and 1500 soles, 19% between 1501 and 2500 soles and only 14% had an income of more than 2500 soles.

**Figure 5:**
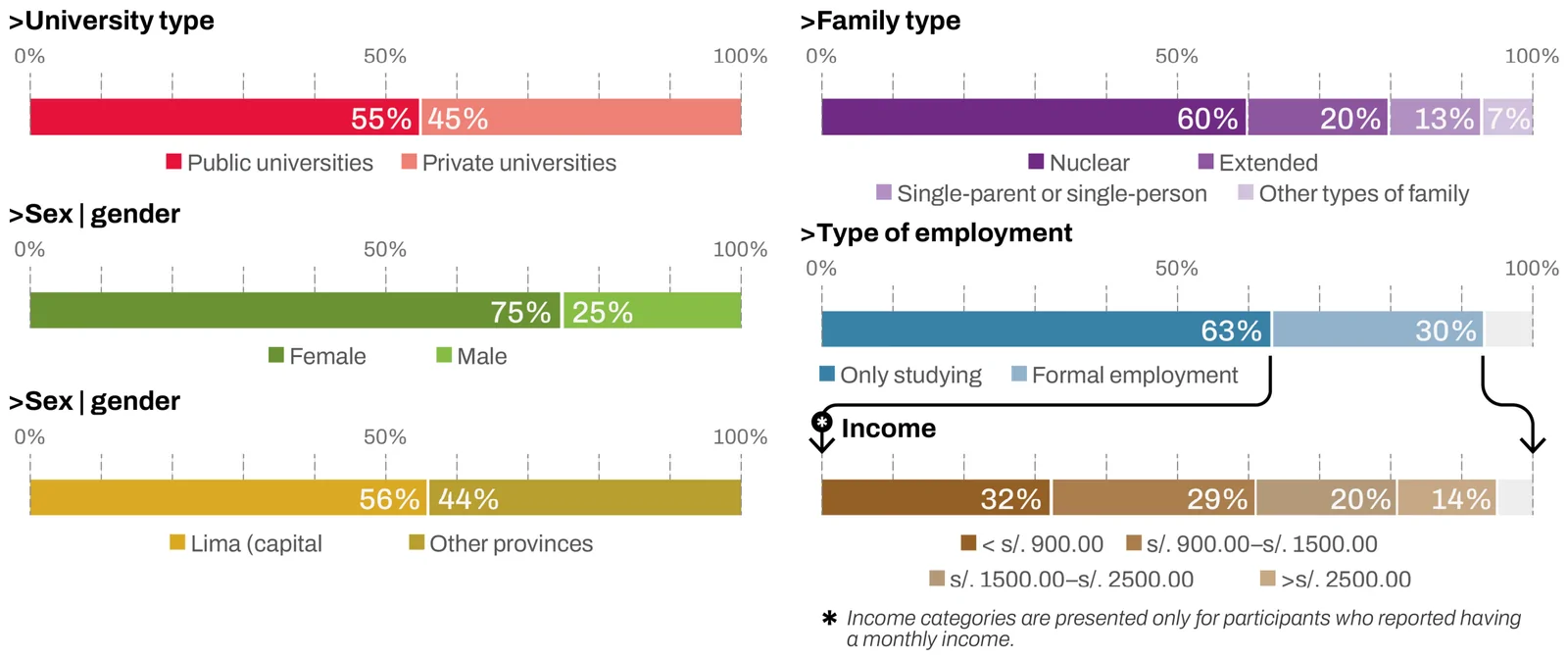
Sociodemographic characteristics of the participants (developed by the authors).

Figure 5 presents a descriptive summary of the study participants based on demographic and socioeconomic variables. It should be noted that these distributions reflect the characteristics of the convenience sample obtained and are not necessarily representative of the broader university student population. The sample shows a predominance of students from public universities and of female gender (approximately 75%), which could suggest disparities in access to private education and a higher female representation in the sample. Additionally, there is a concentration of students from Lima, potentially linked to greater academic opportunities in the capital. Regarding family structure, nuclear families prevail, but there are also relevant percentages of extended and single-parent families, reflecting structural diversity. Finally, the predominantly low-income levels and the prevalence of students without formal employment highlight a vulnerable economic context that could impact their educational and employment opportunities [19].

A convenience sampling strategy was employed, as participants were recruited through the researchers’ existing academic networks. This approach was adopted due to practical constraints of access and feasibility inherent to this pilot study; however, it constitutes a significant methodological limitation, as it introduces selection bias and precludes generalization of findings to the broader university student population in Peru. No probabilistic sampling or formal power analysis was conducted to determine the required sample size, which further restricts the inferential scope of the results. A total of 350 students initially accessed the survey, but 14 did not complete it, resulting in an attrition rate of 4.2% and a final sample of 336 valid responses.

### 2.1. Instruments

As an instrument, a card with items related to social factors was used and a scale was developed based on the Scale to Measure Empowerment for Health Care in Patients with Long-Term Conditions (EECS); this instrument has 51 items developed based on concepts about communication with health professionals, information related to the condition, feelings of control, self-efficacy, coping skills and ability to achieve change. It presents as a response format a 4-point Likert scale of response, from strongly disagree to strongly agree. 

The Scale for Measuring Health Care Empowerment in College Students (ECSU) considered the proposed dimensions and factor structure. Given the need for an instrument applicable to university students without diagnosed diseases, 21 items were eliminated as they were considered directly related to chronic diseases; the instrument now consists of 30 items, 28 positively worded and two negatively worded (item 4 and 21). The positive attitude and sense of control presents 10 items, as do the dimensions of knowledge and confidence in decision making, and empowering others. The response format retains the four-point Likert scale of the original instrument (1 = strongly disagree to 4 = strongly agree). It is acknowledged that a four-point format, by omitting a neutral midpoint, may introduce acquiescence bias, as respondents are required to indicate either agreement or disagreement. Future adaptations of the instrument should consider a five- or seven-point scale to allow for a true neutral response and reduce forced-choice bias.

The adaptation of the ECSU scale followed a content-based approach to ensure its suitability for a non-clinical population. Specifically, the 19 items removed from the original EECS instrument were those explicitly referring to chronic disease management, treatment adherence, and patient–provider interactions in clinical contexts, which were not applicable to university students without diagnosed chronic conditions. The remaining items were reviewed to preserve the conceptual structure of health care empowerment, maintaining the original dimensions of the instrument.

The evidence of content-based validity of the ECSU Scale was estimated based on the judgment of 5 experts. With the information obtained, the Aiken V Coefficient was calculated, for the instrument in general a value of 0.95 and *p* < 0.001 ** was obtained, while each of the dimensions obtained values higher than 0.96 (*p* < 0.001 **). The structure of the original instrument was confirmed. 

For the confirmatory factor analysis (CFA), the maximum likelihood (ML) method was employed. The results showed that the three-factor model had adequate fit indices (CFI = 0.908, TLI = 0.898, RMSEA = 0.061 [0.056, 0.066]), confirming the original three-factor structure of the original instrument. The factor loadings were mostly adequate at >0.30 except for item 5; however, it was decided to keep it since its elimination did not significantly improve the model or the reliability value.

To estimate the evidence of reliability based on internal consistency, Cronbach’s Alpha Coefficient and McDonald’s Omega Coefficient were used, using data from the study sample (336 seniors and graduates). The results evidenced that the scale presents a high internal consistency with a value of α = 0.859 and a ω = 0.835. The positive attitude and desire for control dimension obtained a value of α = 0.880 and a ω = 0.886, the knowledge and confidence in decision making dimension obtained a value of α = 0.877 and a ω = 0.886 and the dimension obtained a value of α = 0.701 and a ω = 0.729.

These results support that the adapted instrument maintains adequate psychometric properties for the study population, although further validation in different populations is recommended.

### 2.2. Procedure

Data collection was conducted online using Google Forms (Google LLC, Mountain View, CA, USA). The link with the instrument was sent via e-mail and WhatsApp to students with whom previous contact was maintained (universities where the researchers worked and former students) as well as to teachers from other universities so that they could share it with their students through these same channels. 

Through a personal message they were invited to participate, explaining in the initial paragraphs the objective of the research. Acceptance to participate voluntarily in the research was requested by selecting the appropriate option in the informed consent form in the second section of the form; the informed consent guaranteed the anonymous and confidential use of the data collected. 

The third section of the instrument presented the instructions and respective items. Participants completed the instrument in a self-administered manner in approximately 15 min.

### 2.3. Data Analysis

For data analysis, IBM SPSS Statistics for Windows, version 19.0 (IBM Corp., Armonk, NY, USA), was used. First, the descriptive analysis was carried out, using the instrument’s scale and establishing the frequencies according to the levels presented by the variable and its dimensions.

To identify the social factors of greater risk in the empowerment for self-care in health in university students, the ordinal logistic regression analysis was used at a 95% confidence level. Given the cross-sectional nature of the study, results are reported as associations rather than causal impacts. Although no a priori power analysis was conducted, a post hoc sensitivity analysis indicated that a sample of 336 participants provides adequate statistical power (≥0.80) to detect small-to-medium effect sizes in ordinal logistic regression at α = 0.05, consistent with methodological recommendations for this type of analysis. This is further supported by the reviewer’s observation that N = 336 meets the minimum requirements for ordinal logistic regression. Nevertheless, the absence of probabilistic sampling limits the external validity of the findings, and results should be interpreted within the scope of this pilot study.

## 3. Results

The corresponding analysis shows that 52% of the university students present a high level of empowerment for health care, 45% a medium level, and a small proportion (3%) at a low level. It is important to highlight that 55% of the students also present a high level in the dimensions knowledge and understanding in decision making, while 49% of students show a moderate level in the dimensions positive attitude and sense of control, being the ‘empowering others’ dimension the one in which a lower percentage of students showed a high level as shown in Table 1. 

### 3.1. Level of Empowerment for Health Care in University Students, According to the Socio-Demographic Variables Analyzed

The descriptive analysis of the health care empowerment variable according to the sociodemographic variables analyzed showed that graduate students have a higher level of empowerment for self-care than undergraduate students; in addition, the level of health care empowerment is slightly higher in students from private universities and in male students (more than 56% reached high levels). It is important to note that the level of empowerment for health care increases as students get older, 70% of students over 30 years old presented high levels of empowerment, while only 50% of students aged 22–30 years old and 30% of students aged 19–21 years old reached a high level of empowerment. The level of empowerment of university students in Lima and the province did not show major differences, with around 50% of students reaching a high level of empowerment. 

**Figure 6:**
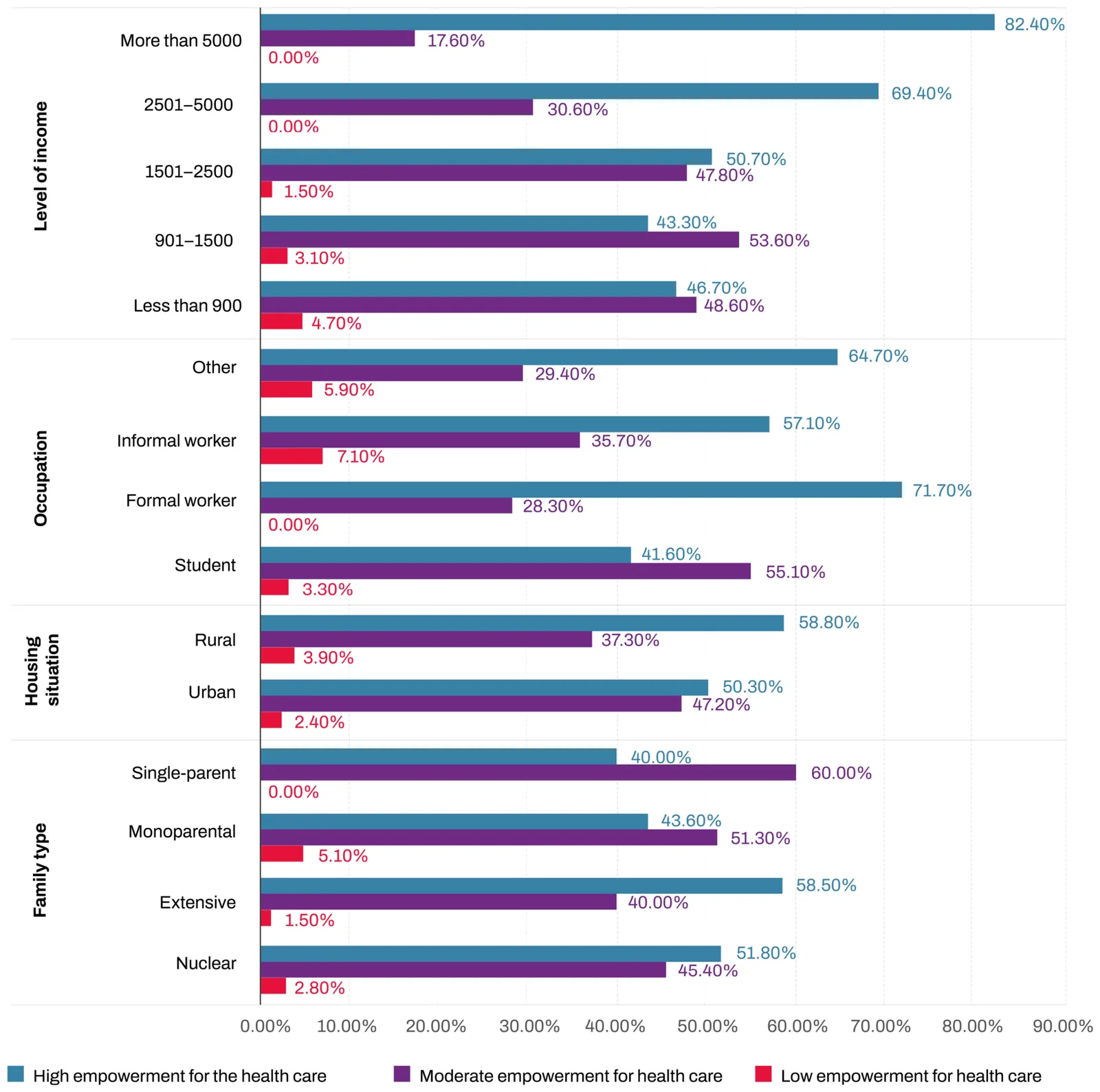
Level and frequency distribution of variable empowerment for health care according to some social variables.

Regarding income level, it was found that about 70% of students whose income was greater than 2500 soles and had a formal job had a high level of empowerment, while 55% of students who were not yet working reached only medium levels of empowerment. Regarding the type of family, it was found that students whose families are extended and nuclear, are those who show the highest level of empowerment for health care (51–58% of students reached high levels respectively), while between 50–60% of students who live alone or with a parent showed moderate levels of empowerment for health care. No major differences were found between urban and rural housing, with just over 50% of both groups showing high levels of empowerment, as shown in Figure 6 (developed by the authors).

### 3.2. Models to Estimate the Highest Risk Factor in Health Care Empowerment

#### 3.2.1. General Hypothesis

The social variables: income level, housing situation, and type of family were examined for their association with health care empowerment in university students. 

The test determined whether the data obtained fit the proposed model, showing that the level of health care empowerment depends on social factors, according to Chi-square = 24.36 and *p* < 0.01 versus statistical significance α = 0.05 (*p*-value < α). The goodness-of-fit test revealed Pearson’s *p*-values and Deviance greater than 5% and the pseudo-R-squared value indicated that 8.8% of the variability is explained by the model, suggesting a limited proportion of explained variance. The Wald statistic revealed significant effects of income level (three categories: <900, 901–1500, and 1501–2500 soles), housing situation (urban), and family type (extended) on empowerment for health care. 

The model is expressed as follows: *P*(Empowerment ≤ j) is the cumulative probability that the empowerment outcome falls at or below category j; α_j_ are the threshold parameters for each response category j; *β*_1_ is the coefficient for income level (NI: coded as three categories: <900, 901–1500, and 1501–2500 soles per month); *β*_2_ is the coefficient for housing situation (HS: 1 = urban, 0 = rural); and *β*_3_ is the coefficient for family type (FT: 1 = extended, 0 = other).

$$P(Empowerment\leq j)=\frac{1}{1+exp\;[-(\alpha_{j}-\beta_{1}NI-\beta_{2}HS-\beta_{3}FT)]}$$

The parallel lines test established that the proposed model meets the parallelism condition (Chi-square = 5.92 with a significance of 0.656); therefore, there is evidence that the Cauchy link function used is appropriate, and the regression coefficients βK are the same among the response categories, thus meeting the parallel lines condition. 

Table 2 shows the models proposed to estimate the highest risk factor in health care empowerment. Three models were proposed, considering income level, housing situation, and family type, as these were the factors that responded to the initially proposed model. It was found that the odds of reaching a low level of health care empowerment when the person has an income level of less than 900 are 1.10 times higher than when the person has an income level of 1501 to 2500 soles, lives in an urban area and belongs to an extended family, and 1.14 times higher if the income level is 901 to 1500 soles, lives in an urban area and belongs to an extended family. 

#### 3.2.2. Specific Hypotheses

Determination of the fit of the data to the model indicated that the level of positive attitude and sense of control depends on social factors (Chi-square = 14.16 and *p*-value = 0.015 versus statistical significance α = 0.05 [*p*-value < α]). The goodness-of-fit test revealed that Pearson’s *p*-values and Deviance are greater than 5.0% and the Pseudo R-squared value indicated that 5.0% of the variability was explained by the model, suggesting a limited proportion of explained variance. 

The Wald statistic used for the individual analysis of the dependent variables revealed that only income level in two categories (less than 900 and 901–1500) and type of university, when this is national were significant at 10%, so it was concluded that they were significantly associated with the level of positive attitude and sense of control of university students.

The model is expressed as follows: P (Positive attitude ≤ j) is the cumulative probability that the positive attitude and sense of control outcome falls at or below category j; α^j^ are the threshold parameters; β_1_ is the coefficient for income level (NI: <900 and 901–1500 soles categories); β_2_ is the coefficient for type of university (TU: 1 = national/public, 0 = private).

$$P(Positive\;attitude\leq j)=\frac{1}{1+exp\;[-(\alpha_{j}-\beta_{1}NI-\beta_{2}TU)]}$$

As in the general hypothesis, the parallel lines test established that the proposed model met the parallelism condition (Chi-square = 4.29 with a significance of 0.509), evidencing that the Cauchy link function used is appropriate and that the regression coefficients βK are the same across response categories. 

Table 3 shows the models proposed to estimate the highest risk factor for the level of positive attitude and sense of control. Two models were proposed, considering the level of income and the type of university, as these were the factors that responded to the model initially proposed. It was found that the odds of reaching a medium level of positive attitude and sense of control when the person has an income level of less than 900 soles and studies in a national university are 1.05 times higher than when the person has an income level of 901 to 1500 soles and studies in a national university, while the odds of reaching a low level of positive attitude and sense of control will be 1.32 times higher for a person who has an income level of 901 to 1500 soles and studies in a private university than for a person with the same income level who studies in a national university. 

The determination of data fit for the model indicates that the level of knowledge and confidence for decision making depends on social factors (Chi-square = 18.41 and *p*-value < 0.001 versus statistical significance α = 0.05 [*p*-value < α]). The goodness-of-fit test revealed Pearson’s *p*-values and Deviance greater than 5% and the pseudo R-squared value indicates that 6.7% of the variability is explained by the model, this value being low [12].

The results of the Wald statistic used for the individual analysis of the independent variables revealed that the significant estimators at 10% were the type of university, when it is national, and the university level when it is undergraduate; therefore, they were significantly associated with the level of knowledge and confidence for decision making [6].

The model is expressed as follows: *P* (Knowledge and confidence ≤ j) is the cumulative probability that the knowledge and confidence for decision-making outcome falls at or below category j; *α^j^* are the threshold parameters; *β*_1_ is the coefficient for type of university (TU: 1 = national/public, 0 = private); *β*_2_ is the coefficient for university level (UL: 1 = undergraduate, 0 = postgraduate).

$$P(Knowledge\;and\;confidence\leq j)=\frac{1}{1+exp\;[-(\alpha_{j}-\beta_{1}TU-\beta_{2}UL)]}$$

The parallel lines test established that the proposed model meets the parallelism condition (Chi-square statistic = 3.244 with a significance of 0.197); therefore, the Cauchy link function used is also adequate, and the regression coefficients βK are the same between response categories [1].

Table 4 shows the models proposed to estimate the highest risk factor, the level of knowledge and confidence for decision making. Two models were proposed, considering the type of university and university level, as these were the factors that responded to the initially proposed model. It was found that the odds of reaching a low level of knowledge and confidence for decision making, when the person studies in a national university and is an undergraduate student are 1.14 times higher than when the person studies in a national university and is a graduate student; it will be 1.19 times higher if the person studies in a national university and is an undergraduate and 1.36 times higher if the person studies in a private university and is an undergraduate [15].

Determination of data fit for the model indicated that the ability to enable others depends on social factors, according to Chi-square = 38.02 and *p*-value < 0.001 versus statistical significance α = 0.05 (*p*-value < α). The goodness-of-fit test revealed that the Pearson and Deviance *p*-values are greater than 5% and the Pseudo R-squared value indicates that 12.8% of the variability is explained by the model, this value being low [17].

The individual analysis of the dependent variables (Wald statistic) that the estimators university levels when undergraduate, the type of family (nuclear and extended), and the occupation of the student (formal worker) were significant at 10% therefore they have an impact on the ability to enable others as can be seen in Table 5.

The model is expressed as follows: *P*(Ability to empower others ≤ j) is the cumulative probability that the ability to empower others outcome falls at or below category j; *α^j^* are the threshold parameters; *β*_1_ is the coefficient for university level (UL: 1 = undergraduate, 0 = postgraduate); *β*_2_ is the coefficient for family type (FT: nuclear or extended vs. other); β_3_ is the coefficient for occupation (Oc: 1 = formal worker, 0 = not formally employed).

$$P(Ability\;to\;empower\;others\leq j)=\frac{1}{1+exp\;[-(\alpha_{j}-\beta_{1}UL-\beta_{2}FT-\beta_{3}Oc)]}$$

In this case, the proposed model also meets the parallel lines condition (Chi-square = 5.247 with a significance of 0.630); therefore, there is evidence that the Cauchy link function used is appropriate, and the regression coefficients βK are the same across response categories. 

Table 5 shows the models proposed to estimate the highest risk factor for the ability to enable others. Three models were proposed, considering university level, type of family and occupation of the student, as they were the factors that responded to the initially proposed model. It was found that the odds of presenting a low capacity to enable others when the person is an undergraduate student, belongs to an extended family and is a formal worker are 1.19 times greater than when the person is a graduate student, belongs to an extended family and is a formal worker; it will be 1.19 times greater if the person is an undergraduate student, belongs to a nuclear family and is a formal worker; while it will be 1.14 times greater if the person is a graduate student, belongs to a nuclear family and is a formal worker [14].

## 4. Discussion

The findings of this pilot study indicate that income level, housing situation, and family type were significantly associated with health care empowerment among the university students sampled [2,15,20]. These were the social determinants most strongly associated with the dependent variable, though given the cross-sectional convenience sample, these associations cannot be interpreted as causal. The income thresholds used in this study (<900, 901–1500, and 1501–2500 soles per month) are contextually relevant: as of 2024, Peru’s statutory minimum wage (Remuneración Mínima Vital) is 1025 soles per month, and the national poverty line is approximately 446 soles per person per month. Accordingly, the lowest income category (<900 soles) represents students living at or below subsistence level, where access to food, transportation, and health services is structurally constrained—conditions that directly limit the material basis for developing health care empowerment. The category of 901–1500 soles falls between the poverty line and the minimum wage, reflecting continued economic precarity. These thresholds therefore represent meaningful structural boundaries, not merely statistical cutpoints. The odds of reaching a low level of health care empowerment were 1.10 times higher among students earning less than 900 soles compared to those earning 1501–2500 soles, living in urban areas and belonging to extended families; and 1.14 times higher among those earning 901–1500 soles under the same conditions [10]. Regarding positive attitude and sense of control, two models were proposed considering income level and type of university [1,8,12].

The odds of reaching a medium level of positive attitude and sense of control were 1.05 times higher among students earning less than 900 soles attending a national university compared to those earning 901–1500 soles at the same institution type, while the odds of reaching a low level were 1.32 times higher among students earning 901–1500 soles at a private university [3]. Regarding parental educational attainment—identified in the literature as a clear social barrier to self-efficacy—this variable was not included in the regression model due to the original instrument design and data collection constraints. However, it is recognized as a theoretically relevant determinant that should be incorporated as an explicit variable in future studies with broader sampling designs. Beyond material resources, the information environment itself may act as an invisible discriminatory boundary: students from lower-income or first-generation backgrounds often have more limited access to reliable health information and digital tools, which constrains the development of health literacy and, in turn, of health care empowerment. From this perspective, income thresholds operate not only as statistical cutpoints but also as barriers that restrict access to the information required for informed self-care, reinforcing existing inequalities in a manner less visible than economic deprivation alone.

The data found on the type of family and empowerment assumes the family as the fundamental nucleus where the idea of self-responsibility in health is introduced; therefore, the family in its role constitutes a non-formal educational agent, a fundamental promoter of healthy habits in the population [2]. The strengthening of healthy and progressive habits through formal and non-formal educational channels and where the configuration of empowerment would help to take control over their lives [5,20,21]. 

It is important to note that the pseudo R^2^ values in ordinal logistic regression are not directly comparable to R^2^ in ordinary least squares regression and typically yield lower magnitudes in social and health science research. The values obtained in this study (5.0–12.8%) indicate that the models capture statistically meaningful associations while acknowledging that health care empowerment is influenced by a broad array of factors beyond those examined here.

These findings are consistent with the broader international literature showing that low socioeconomic status and limited family support are associated with lower self-efficacy and poorer health self-management among university students. Similar associations have been observed in vulnerable populations across Latin America and other regions, where empowerment appears linked to structural inequalities and differential access to educational opportunities [4,12,22,23,24,25].

In addition, it is important to consider that the pseudo R^2^ values obtained in the models ranged between 5% and 12%, indicating a relatively low explanatory power. However, this is common in social and behavioral research, where complex phenomena such as health care empowerment are influenced by multiple unobserved psychological, cultural, and contextual factors. Therefore, the results should be interpreted as statistically significant associations rather than strong predictive relationships. It should also be noted that several odds ratios were close to 1.0 (for example, 1.05 and 1.10), indicating that the practical magnitude of these differences is modest despite reaching statistical significance. Despite this, the identified social variables provide valuable insights into relevant factors associated with variations in health care empowerment. In addition, because public universities in Peru tend to enrol a higher proportion of low-income and first-generation students, type of university and income level are likely to be correlated; the low pseudo-R^2^ values are consistent with residual confounding, and type of university may partly act as a proxy for economic situation rather than an independent risk factor. This possibility of confounding should be considered when interpreting the models.

It is also important to recognize that the cross-sectional and correlational design of this research does not allow establishing causality. Therefore, the results should be interpreted as associations rather than impacts or effects. The study suggests potential relationships between social determinants and empowerment, but longitudinal or experimental designs would be required to confirm causal pathways. From a practical standpoint, low health care empowerment is not a merely theoretical concern: students with limited empowerment are at heightened risk of delaying medical consultations until conditions become severe, experiencing worsening mental health that may lead to academic leave of absence, and ultimately dropping out of school. These outcomes carry long-term consequences for both individual well-being and public health systems. Universities that fail to address the structural conditions underlying low empowerment—such as income insecurity, lack of health insurance, and caregiving responsibilities—may inadvertently reproduce health inequalities among their student populations [26]. Concrete institutional adjustments can make a measurable difference: free or subsidized on-campus health services for low-income students, flexible attendance or online learning options for those with caregiving or work obligations, and targeted health literacy programs for first-generation students would directly address the structural barriers identified in this study. Rather than optional forms of institutional support, these measures should be understood as reasonable accommodations aimed at correcting structural disadvantage and protecting students’ right to equal educational and health opportunities [26,27].

Several limitations of this pilot study must be explicitly acknowledged. First, the convenience sampling strategy introduces selection bias, as participants were recruited through the researchers’ existing academic networks, which may overrepresent students with greater institutional engagement or digital access. Second, the sample exhibits notable demographic imbalances: approximately 75% of participants were female and 56% were from Lima. These proportions may reflect genuine population characteristics of certain university programs, but they may also partly result from the sampling method itself—given that three of the four authors are female, and recruitment relied on personal networks. It is therefore not possible to determine whether the observed empowerment patterns reflect population-level trends or are artifacts of the sample composition. The possible underrepresentation of male students and students from provincial or rural areas limits the applicability of findings across genders and regions. Third, the study covered only five universities from Lima and one from a province, excluding a large portion of Peru’s diverse higher education system. Fourth, the absence of probabilistic sampling and a formal a priori power analysis restricts the inferential scope of the results. Fifth, the four-point Likert response format, which omits a neutral midpoint and forces respondents to indicate either agreement or disagreement, may have introduced acquiescence bias into the empowerment measures. Consequently, the findings cannot be generalized beyond the 336 individuals studied. Future studies should employ probabilistic sampling with adequate power and include broader geographical and institutional representation to establish external validity. Despite these limitations, this pilot study provides preliminary exploratory evidence on an underexplored topic in the Peruvian and Latin American context, and serves as a basis for designing more rigorous future research.

## 5. Conclusions

Health care empowerment among university students was examined in this pilot study. Within the convenience sample analyzed, lower income levels, enrollment in public universities, and belonging to extended family structures were associated with lower levels of empowerment. These associations suggest that structural inequalities may be linked to students’ capacity for self-management, decision-making, and agency in health-related contexts, though causal conclusions cannot be drawn from this cross-sectional design.

Rather than operating independently, these determinants appear to interact in complex ways, associated with distinct empowerment profiles within the student population. For instance, the combination of low socioeconomic status and limited institutional support may be linked to reduced access to health information and weaker perceived control, both of which are critical dimensions of empowerment such as self-efficacy and the ability to influence others. These exploratory findings are consistent with broader theoretical perspectives that conceptualize empowerment as a context-dependent and socially mediated process.

Conversely, higher income levels, postgraduate status, and enrollment in private institutions were associated with higher levels of empowerment, likely reflecting greater access to resources, stronger support networks, and enhanced opportunities for informed decision-making. This pattern reinforces the idea that empowerment is not solely an individual attribute but is strongly conditioned by structural and institutional factors.

From a practical standpoint, these results highlight the need for universities to move beyond their traditional academic role and adopt a more active position in promoting student health. This involves designing context-sensitive interventions aimed at reducing socioeconomic barriers, strengthening health literacy, and fostering environments that support autonomy and self-care.

As a pilot study based on a convenience sample, these findings are exploratory in nature and cannot be generalized beyond the 336 participants studied. The cross-sectional and non-probabilistic design limits causal inference and generalizability. Future research should employ probabilistic sampling, balanced demographic representation, and longitudinal or mixed-methods designs, including structural equation modelling (SEM) to map the interrelations among the many unobserved factors, to better understand the dynamic processes through which social determinants are associated with health care empowerment over time. This pilot study is intended as a model for such future investigations, correctly implemented with adequate sample size and sampling rigor.

## Figures and Tables

**Table 1:** Level and frequency distribution of the variable empowerment for health care and its dimensions.

Level	Empowerment for Health Care		Positive Attitude and Sense of Control		Knowledge and Understanding in Decision Making		Enabling Others	
	fr	%	fr	%	fr	%	fr	%
Low	9	3	19	6	10	3	19	7
Moderate	154	45	165	49	140	42	183	54
High	174	52	152	45	187	55	130	39
Total	337	100	337	100	337	100	337	100

**Table 2:** Probability of occurrence and odds ratio (OR) of the ordinal regression model of health care empowerment and social factors.

Dependent Variable	Probability of Occurrence	Odds Ratio (OR)
Model Y ≤ j/income level less than 900, urban housing situation, extended family type.		
Y = low empowerment	<0.01	
Y = medium empowerment	0.49	
Y = high empowerment	0.51	Rj = 3|Ni = 3.1| = 1.10
Model Y ≤ j/income level 901–1500, urban living situation, extended family type.		
Y = low empowerment	0.000012261	
Y = medium empowerment	0.51	
Y = high empowerment	0.49	Rj = 3|Ni = 3.2 = 1.14
Model Y ≤ j/income level 1501–2500, urban living situation, extended family type.		
Y = low empowerment	0.000007253	
Y = medium empowerment	0.44	
Y = high empowerment	0.56	

**Table 3:** Probability of occurrence and odds ratio (OR) of the ordinal regression model of positive attitude and sense of control, and social factors.

Dependent Variable	Probability of Occurrence	Odds Ratio (OR)
Model Y ≤ j/income level less than 900, national university.		
Y = positive attitude and low sense of control	<0.01	
Y = positive attitude and medium sense of control	0.62	
Y = positive attitude and high sense of control	0.37	Rj = 2|Ni = 2.1) = 1.05
Model Y ≤ j/income level of 901–1500, national university.		
Y = positive attitude and low sense of control	<0.01	
Y = positive attitude and medium sense of control	0.61	
Y = positive attitude and high sense of control	0.34	Rj = 3|TU = 2.1 = 1.32

**Table 4:** Probability of occurrence and odds ratio (OR) of the ordinal regression model, knowledge and confidence for decision making, and social factors.

Dependent Variable	Probability of Occurrence	Odds Ratio (OR)
Model Y ≤ j/national university, undergraduate		
Y = low knowledge and confidence in decision making	<0.01	
Y = medium knowledge and confidence in decision making	0.56	
Y = high knowledge of and confidence in decision making	0.44	Rj = 3|Ni = 2.1 = 1.19
Model Y ≤ j/private university, undergraduate		
Y = low knowledge and confidence in decision making	<0.01	
Y = medium knowledge and confidence in decision making	0.45	
Y = high knowledge of and confidence in decision making	0.55	Rj = 3|Ni = 2.1 = 1.36
Model Y ≤ j/national university, postgraduate		
Y = low knowledge and confidence in decision making	<0.01	
Y = medium knowledge and confidence in decision making	0.35	
Y = high knowledge of and confidence in decision making	0.65	Rj = 3|TU = 2.1 = 1.143
Model Y ≤ j/national university, postgraduate		
Y = low knowledge and confidence in decision making	<0.01	
Y = medium knowledge and confidence in decision making	0.19	
Y = high knowledge of and confidence in decision making	0.75	

**Table 5:** Probability of occurrence and odds ratio (OR) of the ordinal regression model of the ability to empower others and social factors.

Dependent Variable	Probability of Occurrence	Odds Ratio (OR)
Model Y ≤ j/undergraduate, extended family and formal worker.		
Y = ability to enable others low	<0.01	
Y = ability to enable others medium	0.66	
Y = ability to enable others high	0.48	Rj = 3|Ni = 2.1) = 1.19
Model Y ≤ j/, undergraduate, nuclear family and formal worker.		
Y = ability to enable others low	<0.01	
Y = ability to enable others medium	0.50	
Y = ability to enable others high	0.59	Rj = 3|Ni = 2.1 = 1.19
Model Y ≤ j/postgraduate, nuclear family and formal worker.		
Y = ability to enable others low	<0.01	
Y = ability to enable others medium	0.37	
Y = ability to enable others high	0.55	Rj = 3|TU = 2.1 = 1.14
Model Y ≤ j/postgraduate, extended family and formal worker.		
Y = ability to enable others low	<0.01	
Y = ability to enable others medium	0.19	
Y = ability to enable others high	0.65	

## Data Availability

The original contributions presented in this study are included in the article. Further inquiries can be directed to the corresponding author.

## References

[1] Blas E., Kurup A. (2021). Equity, Social Determinants and Public Health Programmes.

[2] Centers for Disease Control and Prevention (2014). Social Determinants of Health: Know What Affects Health. https://www.cdc.gov/socialdeterminants.

[3] Fernández-Peña J., Suárez Orozco C. (2023). Educación y empoderamiento en salud. Bol. Méd. UASLP.

[4] García M., Yunes J., Delgado J. (2023). Factores socioeconómicos y salud en estudiantes. Rev. Médica Chile.

[5] Gómez-Camacho R., Ortiz-Álvarez J. (2023). Vulnerabilidad y determinantes sociales. Salud Pública México.

[6] Holman D., Walker P. (2022). Gender inequalities in health and aging. Aging Health.

[7] Htun N.M.M., Hnin Z.L., Khaing W. (2021). Empowerment and health care access barriers among currently married women in Myanmar. BMC Public Health.

[8] Huston A.C., Bentley A.C. (2010). Human development in societal context. Annu. Rev. Psychol..

[9] Kondo N., Subramanian S.V., Kawachi I. (2021). Economic recession and health inequalities. Am. J. Public Health.

[10] Leal-Soto F., Pérez-Suarez T. (2022). Barreras sociales y empoderamiento. Rev. Médica Chile.

[11] López-Cervantes M., Doubova S.V., Pelcastre-Villafuerte B.E., Serván-Mori E. (2022). Vulnerability and barriers of access to health services. Salud Pública México.

[12] López-Fernández L., Martínez A., Pérez C. (2023). Impacto de los determinantes sociales en adolescentes. Rev. Salud Pública.

[13] Lund C., Brooke-Sumner C., Baingana F., Baron E.C., Breuer E., Chandra P., Haushofer J., Herrman H., Jordans M., Kieling C. (2018). Social determinants of mental disorders and the SDGs. Lancet Psychiatry.

[14] Marmot M. (2021). Social determinants of health inequalities. Lancet.

[15] Ortega-López L., Díaz-Valdés A. (2021). Empoderamiento femenino y salud. Rev. Chil. Salud Pública.

[16] Pais S.C., Rodrigues M., Menezes I. (2014). Community as locus for health formal and non-formal education. Front. Public Health.

[17] Bernal-Ordoñez L.K., NIño-Gutiérrez E.L., Casanova M.L., Treviño del Campo F., Rodríguez A., Jiménez García D.A. (2024). Participación y empoderamiento comunitario en la atención primaria en salud en América Latina: Revisión sistemática exploratoria. Rev. Panam. Salud Pública.

[18] Rodríguez C., Vargas E. (2023). Desarrollo local y empoderamiento. Rev. Salud Pública.

[19] Ruiz-Aquino M., Díaz M. (2021). Patrones de estilo de vida de los estudiantes ingresantes a la Universidad de Huánuco. Rev. Salud UDH.

[20] Ruiz-Aquino M., Echevarría J., Huanca W.E. (2021). Conductas de autocuidado de la salud en estudiantes universitarios. Socialium.

[21] Salcedo M. (2018). El Enfoque de Promoción de la Salud Aplicado en el Currículo. Doctoral Dissertation.

[22] Schrecker T., Bambra C. (2021). Neoliberal epidemics: How politics makes us sick. BMJ.

[23] Oswald T.K., Nguyen M.T., Mirza L., Lund C., Jones H.G., Crowley G., Aslanyan D., Dean K., Schofield P., Hotopf M. (2024). Interventions targeting social determinants of mental disorders and the Sustainable Development Goals: A systematic review of reviews. Psychol. Med..

[24] Whitehead M. (1992). The concepts and principles of equity and health. Int. J. Health Serv..

[25] Williams D.R., Mohammed S.A. (2013). Racism and health: Pathways and scientific evidence. Am. Behav. Sci..

[26] World Health Organization (2008). Closing the Gap in a Generation: Health Equity Through Action on the Social Determinants of Health. https://apps.who.int/iris/handle/10665/43943.

[27] World Health Organization (2022). Social Determinants of Health: Monitoring, Assessment and Policy Interventions. https://www.who.int.

